# Adhesion of Leukocytes to Cerebral Venules Precedes Neuronal Cell Death and Is Sufficient to Trigger Tissue Damage After Cerebral Ischemia

**DOI:** 10.3389/fneur.2021.807658

**Published:** 2022-01-24

**Authors:** Rebecca Isabella Sienel, Hiroharu Kataoka, Seong-Woong Kim, Fatma Burcu Seker, Nikolaus Plesnila

**Affiliations:** ^1^Laboratory of Experimental Stroke Research, Institute for Stroke and Dementia Research (ISD), University of Munich Medical Center, Munich, Germany; ^2^Munich Cluster of Systems Neurology (Synergy), Munich, Germany; ^3^Department of Neurosurgery, Graduate School of Medicine, Kyoto University, Kyoto, Japan; ^4^Department of Neurosurgery, University of Giessen, Giessen, Germany

**Keywords:** ischemic stroke, cerebral vessels, inflammation, leukocytes, adhesion molecules

## Abstract

**Background:**

Leukocytes contribute to tissue damage after cerebral ischemia; however, the mechanisms underlying this process are still unclear. This study investigates the temporal and spatial relationship between vascular leukocyte recruitment and tissue damage and aims to uncover which step of the leukocyte recruitment cascade is involved in ischemic brain injury.

**Methods:**

Male wild-type, ICAM-1-deficient, anti-CD18 antibody treated, or selectin-deficient [fucusyltransferase (FucT IV/VII^−/−^)] mice were subjected to 60 min of middle cerebral artery occlusion (MCAo). The interaction between leukocytes and the cerebrovascular endothelium was quantified by *in vivo* fluorescence microscopy up to 15 h thereafter. Temporal dynamics of neuronal cell death and leukocyte migration were assessed at the same time points and in the same tissue volume by histology.

**Results:**

In wild-type mice, leukocytes started to firmly adhere to the wall of pial postcapillary venules two hours after reperfusion. Three hours later, neuronal loss started and 13 h later, leukocytes transmigrated into brain tissue. Loss of selectin function did not influence this process. Application of an anti-CD18 antibody or genetic deletion of ICAM-1, however, significantly reduced tight adhesion of leukocytes to the cerebrovascular endothelium (-60%; *p* < 0.01) and increased the number of viable neurons in the ischemic penumbra by 5-fold (*p* < 0.01); the number of intraparenchymal leukocytes was not affected.

**Conclusions:**

Our findings suggest that ischemia triggers only a transient adhesion of leukocytes to the venous endothelium and that inhibition of this process is sufficient to partly prevent ischemic tissue damage.

## Introduction

Ischemic stroke is one of the most frequent causes of death and disability worldwide (1, 2). Current therapies include thrombolysis with rtPA or rtPA in combination with mechanical thrombectomy (3–5); however, only up to 30% of stroke patients are eligible for these interventions (5). Hence, there is an ongoing and urgent need for the development of novel therapeutic options for the 70% of stroke patients who do not receive any causal treatment.

For more than four decades, inflammation has been recognized as a major pathomechanism, which is responsible for brain injury following ischemic stroke (6–11). A plethora of elegant experimental and clinical studies discovered that ischemia triggers an acute innate immune response within the brain parenchyma which results in the production of inflammatory cytokines, the upregulation of adhesion molecules on endothelial cells, and the subsequent recruitment of granulocytes and monocytes into ischemic tissue within the first few hours and days after vessel occlusion (12–19). Later on, T-lymphocytes invade the infarcted tissue and may cause further damage (20–22). Despite these impressive steps forward in our understanding of postischemic inflammation, none of the above-mentioned mechanisms translated into a viable therapeutic approach for stroke patients (14). Hence, reevaluation of previous experimental findings and identification of significant knowledge gaps may help identify so far unexplored or neglected therapeutic principles related to postischemic leukocyte recruitment.

Histopathological studies in human tissue, nonhuman primates, and rodents and investigations using radioactively labeled leukocytes in humans and experimental animal models univocally demonstrate that cerebral ischemia is associated with accumulation of polymorphonuclear leukocytes (PMNs) or granulocytes in the brain (10, 11, 23–31). According to experimental studies using radioactively labeled leukocytes or direct visualization of leukocytes by intravital microscopy, recruitment of leukocytes to the ischemic brain starts within the first 2 h after the onset of ischemia (9, 32–35), a time course also supported by investigations in stroke patients (30, 36). The nature of this accumulation seems to follow two different mechanisms: leukocytes may plug capillaries and arterioles during ischemia, thus participating in the so-called no reflow phenomenon (33, 37, 38), and/or they may adhere to the endothelium of postcapillary venules due to upregulation of adhesion molecules (27, 32, 34, 35). No matter which concept of accumulation is followed, most laboratories report that depletion of granulocytes or inhibition or deletion of adhesion molecules reduces ischemic brain damage and improves outcome following experimental stroke (27, 33, 37, 39–50). Hence, there is general agreement that leukocytes accumulate in the brain within the first few hours after cerebral ischemia and inhibition or deletion of adhesion molecules reduce ischemic brain damage. Beyond this generally accepted concept, however, many crucial issues on the role of leukocytes for ischemic tissue damage are still unsolved or highly debated (17, 27, 28). One of the main reasons for this discussion is that most of the above-cited studies used static, histopathological techniques to investigate leukocytes after stroke. Therefore, our knowledge about the dynamics of adhesion, transmigration, and accumulation of leukocytes in the brain after a stroke and how these processes are related to tissue damage is still surprisingly limited.

Technically, leukocyte dynamics after stroke can be addressed by longitudinal *in vivo* imaging; however, the few studies using this approach either focused on the very first hours after cerebral ischemia, a time when the neuronal injury was not yet present, or on time points later than 24 h after stroke, when the ischemic injury had already occurred (27, 32–35, 51). Consequently, we still do not know whether leukocytes are present in the brain when ischemic damage occurs or whether leukocytes are present at the site of injury (6, 7, 17, 19–21, 52–57). To answer these two important issues, we investigated the full-time course and sequence of leukocyte accumulation to the ischemic brain in parallel with neuronal cell death and tried to decipher which part of the leukocyte adhesion cascade may be involved in ischemic tissue damage.

## Materials and Methods

### Ethics Approval

All procedures were reviewed and approved by the respective institutional and governmental authorities and performed according to all regulations.

### Animals

Male 129/Sv mice (129/SvPaslcoCrIBR; 22–26 g body weight; Charles River laboratories), α(1,3) fucosyltransferase IV and VII double knockout [fucusyltransferase (FucT IV^−/−^/VII^−/−^)] mice initially generated by John Lowe and coworkers (58, 59), ICAM-1^−/−^ mice (B6129S4-Icam1^tm1Jcgr^/J; 6–10 weeks, weight 25–30 g, Jackson Laboratories), and male C57BL/6 mice (6–8 weeks, weight 24–26 g; Charles River Laboratories) were used for this study. Genotypes of these mice were confirmed by polymerase chain reaction (PCR) amplification of genomic DNA (Table 1).

**Table 1:** Primers for genotyping.

**Target gene**	**Seuqence**
FucT-IV-/-	5′-CTGGACCGCGTTGACCACCTTCATCTGCTG-3′
	5′-CTGGACCGCGTTGACCACCTTCATCTGCTG-3′
	5′-CGGGACCTCTGGCATCCAAGAGCAGGGGGA-3′
FucT-VII-/-	5′-CCTCTCTCTGGGCCCACATCCCCACTACCG-3′
	5′-GGACTGGCTG CTATTGGGCGAAGTG-3′
	5′-GACGTGGTAGACACGGGCGATGGGAATG AA-3′

All experimental procedures were reviewed and approved by the Animal Ethics Board of the Government of Upper Bavaria. Mice were randomly assigned to experimental groups by drawing lots. All experimental procedures and analyses were performed by a researcher blinded to group allocation or genotype and reported according to the ARRIVE criteria.

### Transient Focal Cerebral Ischemia

Transient focal cerebral ischemia was performed as previously described (60–64). Briefly, animals received buprenorphine for analgesia 30 min before surgery (0.1 mg/kg) and were anesthetized with 1.4–1.8% isoflurane under the tight control of rectal temperature (37°C ± 0.1°C) with a feedback-controlled heating pad. Regional cerebral blood flow (rCBF) over the territory of the middle cerebral artery (MCA) was monitored using laser Doppler fluxmetry (Perimed, Stockholm, Sweden). To occlude the MCA, a silicone-coated monofilament (Doccol Corporation, USA) was introduced into the left common carotid artery and advanced toward the Circle of Willis until a drop of rCBF below 20% of baseline indicated occlusion of the MCA. In sham-operated control animals, the filament was advanced into the circle of Willis without occlusion of the MCA. Thereafter, animals were allowed to wake up. After sixty min, animals were reanesthetized and the filament was removed to allow reperfusion.

### *In vivo* Microscopy

At different time points after focal cerebral ischemia (45 min, 2.5, 4.5, 8.5, or 14.5 h), *in vivo* fluorescence microscopy was performed for up to 2 h as described previously (32, 62, 65–72). Epifluorescence microscopy (EFM) using a CCD camera was used to detect fast processes, such as leukocyte rolling in pial vessels, whereas deep tissue penetrating two-photon scanning microscopy (2-PM; Zeiss LSM 7, Zeiss, Oberkochen Germany) was used to visualize penetrating arterioles and capillaries within the brain parenchyma. Briefly, animals were anesthetized with medetomidine (0.5 mg/kg), midazolam (5 mg/kg), and fentanyl (0.05 mg/kg) i.p., intubated, and mechanically ventilated under continuous recording of end-tidal pCO_2_ using a microcapnometer (Hugo Sachs Elektronik, Hugstetten, Germany). A catheter was implanted into the left femoral artery for continuous blood pressure recordings and fluid management. An acute cranial window was implanted prior to the imaging leaving the dura mater intact (2.0 × 2.0 mm for EFM and 4.0 × 4.0 mm for 2-PM; 2.2 mm lateral and 1.6 mm caudal of bregma). The window was prepared above the medial rim of the MCA territory to allow imaging of the infarct core (no flow) and the adjacent ischemic penumbra (reduced flow). Next, a cover glass (Warner Instruments, Holliston, MA, USA) with the exact size of the craniotomy was placed on top of the exposed dura mater and fixed with bone cement. Finally, a titanium ring was glued around the cover glass. The ring was used to fix the head of the mouse under the intravital microscope and to keep a pool of water between the cover glass and the water immersion objective (Plan Apochromat, NA 1.0; Zeiss, Oberkochen, Germany) used for *in vivo* imaging. The cerebral microvasculature and intravascular leukocytes were visualized by an intravenous injection of 0.5% FITC-dextran (2,000 kDa, Sigma Aldrich, Deisenhofen, Germany) or 0.5% rhodamine 6G (MW 479.01 kDa, Sigma Aldrich, Deisenhofen, Germany), respectively. Rolling leukocytes were defined by their multiple intermittent contacts with the vascular endothelium, thereby leaving the center flow of the vessel. Adherent leukocytes were defined by their firm attachment to the vascular wall for more than 20 s. Anesthesia was maintained by hourly injections of one-third of the dose necessary for the induction of anesthesia. Blood gases and electrolytes were assessed at the end of each experiment. For each experimental group, *n* = 12 wild-type animals were investigated, that is, 120 animals in total (ischemia and reperfusion or sham surgery investigated at five-time different points).

For experiments using 2-photon microscopy, a group subjected to MCAo (*n* = 8) and a sham-operated group (*n* = 8) were investigated 4–6 h after surgery, that is at the time point of the most pronounced interaction of leukocytes with the endothelium of cerebral venules. Due to the reduced acquisition speed of the 2-PM system, adherent leukocytes were defined by their firm attachment to the vascular wall for more than 5 min.

For experiments on transgenic animals (FucT IV^−/−^/VII^−/−^ or ICAM-1^−/−^), *n* = 5 adhesion molecule deficient and *n* = 5 wild-type mice were imaged, that is, a total of 20 mice.

For experiments using anti-CD18 antibodies, *n* = 12 animals were imaged per group, that is, 36 mice in total (untreated, IgG-treated, and anti-CD18 treated).

Altogether, a total of 192 mice were investigated by *in vivo* fluorescence microscopy.

### Inhibition of Leukocyte–Endothelium Interaction (LEIs)

To modulate the interaction of leukocytes with the cerebrovascular endothelium, a rat antimouse anti-CD18 antibody (GAME-46, BD Biosciences, Franklin Lakes, USA) or an isotype-matched control IgG1 (BD Biosciences; Franklin Lakes, USA) was injected into the left internal carotid artery immediately after reperfusion. Doses of GAME-46 and control IgG were the same as those used previously for the attenuation of neutrophil adhesion in mice (1.2 μg/g body weight) (73).

### Histological Evaluation of Neuronal Cell Death and Infarct Volumes

At the end of each *in vivo* microscopy experiment, animals were sacrificed and perfused with 4% paraformaldehyde in phosphate-buffered saline. Brains were harvested and embedded in paraffin, and serial coronal sections (5 μm) were prepared from the area of the brain located underneath the cranial window used for *in vivo* microscopy to allow for coregistration of histological and *in vivo* microscopy data (**Figure 4A**). After staining with hematoxylin–eosin (H&E), three sections were chosen by morphological comparison with a stereological mouse brain atlas (Paxinos and Keith: The Mouse Brain in Stereotaxic Coordinates, 2nd ed. 2004, Academic Press) to match precisely the following coordinates: 2.6 mm dorsal of bregma (midline of the cranial window), 2.1 mm dorsal of bregma (0.5 mm rostral of the midline of the cranial window), and 3.1 mm dorsal of bregma (0.5 mm dorsal of the middle of the cranial window). In these three sections, the number of normal neurons was counted just underneath the cranial window at a depth where leukocyte–endothelium interactions (LEIs) were previously visualized by fluorescence microscopy, that is, up to 400 μm below the cortical surface. The region of interest for counting of neurons was 2.5 mm lateral to the midline and had a size of 0.32 mm × 0.24 mm. As a control, cells were also counted in the same area of the contralateral hemisphere. To investigate neuronal cell death also at a time point when the infarct already matured, two additional groups (MCAo and sham surgery) were investigated at 24 h. Twelve animals were examined per group, that is, 120 animals in total (MCAo or sham surgery investigated at five-time different points).

Infarct volumes or neuronal counts in transgenic mice or in mice receiving anti-CD18 treatment were assessed 24 h after MCAo or sham surgery in 5–15 mice per group.

### Immunohistochemical Detection of Leukocytes

Infiltration of leukocytes into postischemic tissue was examined by immunohistochemistry in brain sections adjacent to those used for the quantification of cell death in wild-type mice (see above). After deparaffinization and blocking of endogenous peroxidase activity with 0.3% H_2_O_2_, sections were incubated with a rat antimouse CD45 polyclonal antibody (DB Biosciences Pharmingen, San Jose, CA, U.S.A.), followed by the incubation with a goat antirat biotinylated secondary antibody. After incubation with a streptavidin-conjugated horseradish peroxidase, antibody binding was visualized with 3,3'-diaminobendidine (DAB). The number of CD45-positive cells was counted in the same region of interest as used for the quantification of cell death (see above).

### Statistical Analysis

The data were tested for normal distribution using the Shapiro–Wilk test. For normally distributed data, differences between two groups were assessed with Student's *t*-test. For not normally distributed data, the Mann–Whitney *U* test was used for the analysis of differences between two groups, and the Friedman one-way analysis of variance on ranks followed by the Student–Newman–Keuls test was used for analyzing differences over time. Data are presented as individual values and as means ± standard deviation (SD). A statistically significant difference between groups was assumed at *p* < 0.05.

## Results

### Physiological Parameters

Mean arterial blood pressure (MABP), pH, pCO_2_, and pO_2_ were within the physiological range at the end of each experiment and did not differ between mice subjected to cerebral ischemia or sham surgery (Table 2).

**Table 2:** Physiological parameters.

			**Time after reperfusion**				
**Paramter**	**Group**	**During MCAo**	**40 min**	**60 min**	**90 min**	**120 min**	**150 min**
MABP (mmHg)	Ischemic	86.0 ± 13.5	90.5 ± 5.6	87.0 ±6.3	84.6 ± 15.5	85.6 ± 3.0	83.5 ± 6.4
	Control	87.7 ± 11.0	87.9 ±	89.6 ± 16.0	85.88 ± 4.5	83.7 ± 14.6	82.6 ± 14.8
pH	Ischemic	7.37 ± 0.02					7.32 ± 0.03
	Control	7.38 ± 0.02					7.32 ± 0.02
PO_2_ (mmHg)	Ischemic	165.9 ± 14.5					151.6 ± 16.8
	Control	165.7 ± 13.8					138.5 ± 16.1
PCO_2_ (mmHg)	Ischemic	38.0 ± 2.0					35.0 ± 3.5
	Control	37.9 ± 1.5					34.6 ± 1.2
Arteriolar diameter (μm)	Ischemic		29.8 ± 1.2	29.3 ± 1.1	29.4 ± 1.3	29.5 ± 1.4	29.7 ± 1.5
	Control		29.1 ± 1.8	28.9 ± 1.4	29.2 ± 1.7	29.0 ± 1.4	29.2 ± 1.5
Venular diameter (μm)	Ischemic		29.4± 1.9	29.5 ± 2.1	29.6 ± 2.1	29.3 ± 1.8	29.7 ± 1.9
	Control		29.2 ± 1.6	28.9 ± 1.6	28.8 ± 1.4	29.1 ± 1.6	29.2 ± 1.7
Capillary density (cm/cm^2^)	Ischemic		138.8 ± 16.6	138.7 ± 15.6	139.8 ± 15.3	139.9 ± 18.0	140.1 ± 15.2
	Control		140.0 ± 15.8	140.7 ± 15.5	139.1 ± 14.6	142.0 ± 15.3	140.1 ± 15.9

*Blood gas and MABP data during MCAo were recorded in 6 animals*.

*Due to catheter clogging data at 150 min could be observed in 5 animals only. Data are given as means ±SD*.

### Leukocyte-Endothelium Interactions (LEIs) Peak 5 h After Focal Cerebral Ischemia

**Figure 1:**
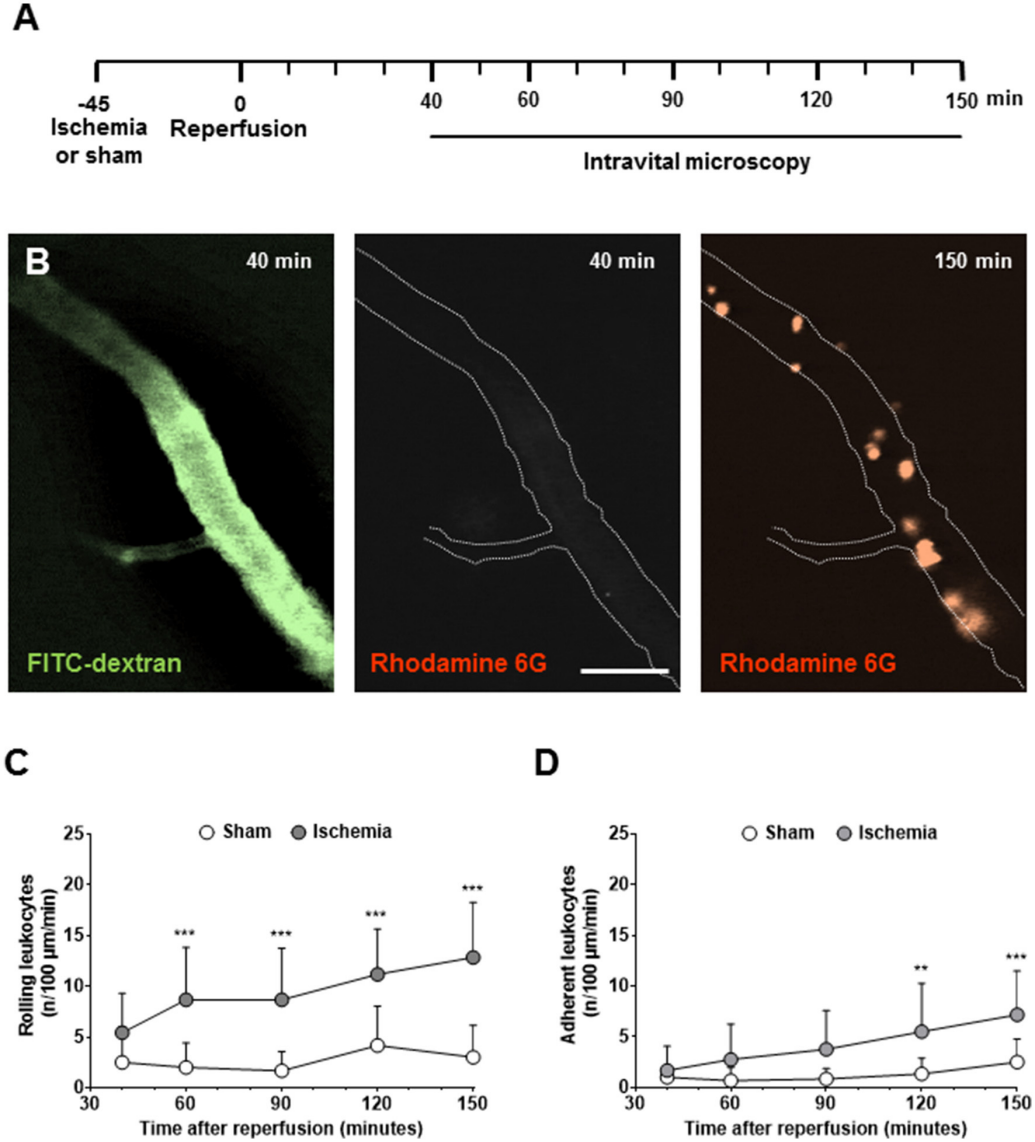
Leukocyte-endothelium interactions (LEIs) in pial venules early after cerebral ischemia. **(A)** Experimental design. Animals were kept in anesthesia during the whole duration of the experiment. To avoid phototoxicity, intravital microscopy was performed 40, 60, 90, 120, and 150 min after reperfusion for only one min. **(B)** Representative images of pial venules 40 and 150 min after 60 min of ischemia (size bar: 50 μm). Perfused vessels are visualized with the plasma marker FITC-dextran, leukocytes with rhodamine 6G. While no leukocytes are present 40 min after reperfusion, a large number of LEIs are visible 90 min later. Quantification of the number of leukocytes rolling **(C)** or sticking **(D)** to the endothelium of pial venules. Two-way ANOVA: **p < 0.01, ***p < 0.001 vs. sham. Means ± SD of 12 animals per group.

When investigating LEIs 40–150 min after reperfusion from 60-min MCAo (Figure 1A) in the pial vessel by staining the vessel lumen with FITC-dextran and leukocytes with rhodamine 6G, almost no interactions were observed 40 min after reperfusion, whereas large numbers of leukocytes were present 90 min thereafter (Figure 1B). LEI occurred exclusively in postcapillary venules; significant interactions with the endothelium of pial arterioles were never observed (data not shown). When quantifying these observations, only physiological rolling (<5 cells/100 μm/min) was observed in sham-operated and ischemic animals within the first hour of reperfusion (Figure 1C). Thereafter, significantly increased rolling and adhesion occurred only in mice subjected to cerebral ischemia (Figures 1C,D). At the end of the observation time, that is, 2.5 h after reperfusion, rolling and adhesion were significantly increased in mice subjected to ischemic stroke (Figures 1C,D; *p* < 0.001).

**Figure 2:**
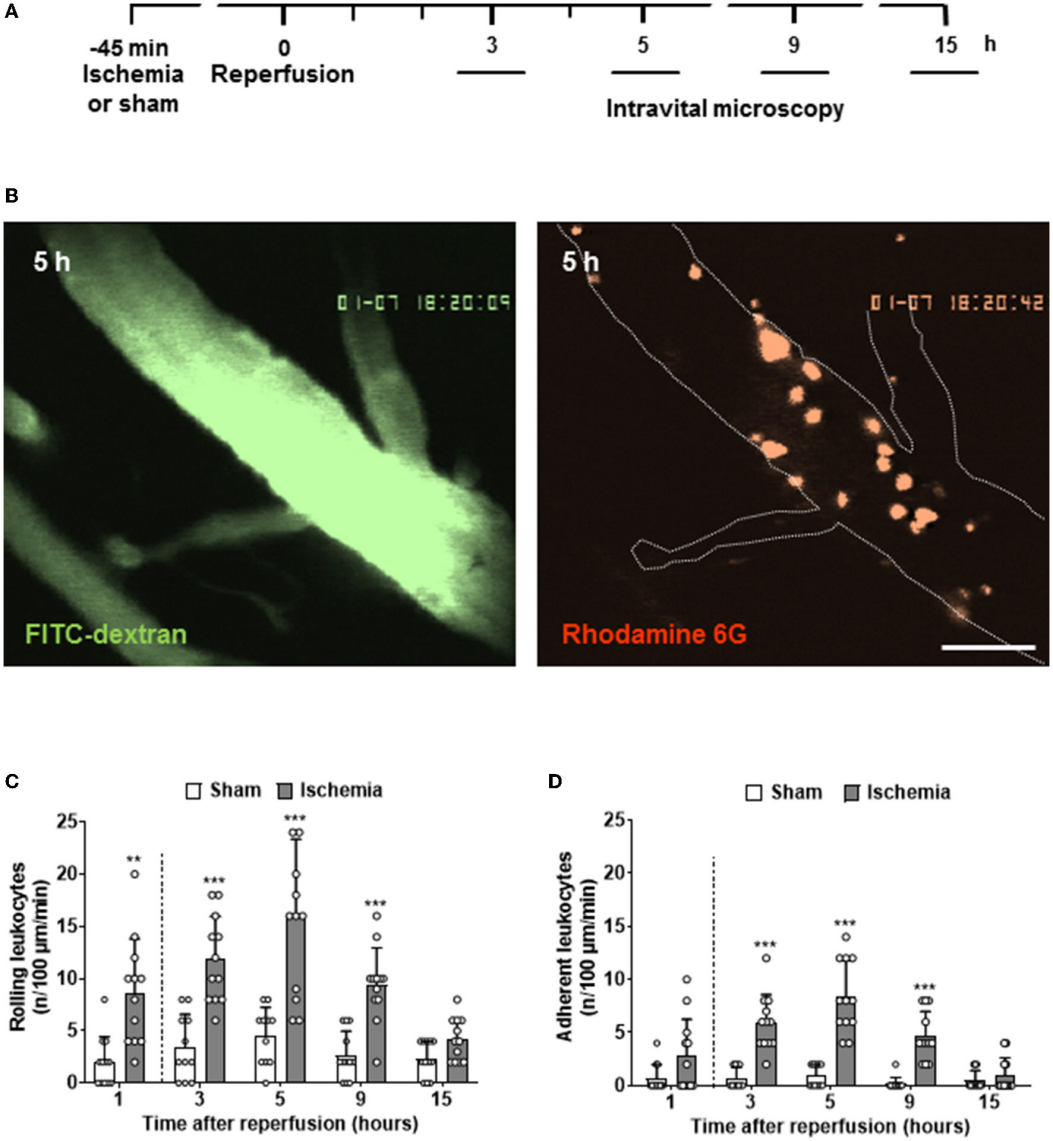
Full time course of LEIs in pial venules following cerebral ischemia. **(A)** Experimental design. Different groups of animals (*n* = 12 each) were imaged 3, 5, 9, and 15 h after reperfusion for one min. **(B)** Representative image of a large pial venule visualized with the plasma marker FITC-dextran five hours after reperfusion (left; size bar: 50 μm). A large number of adherent, rhodamine 6G-labled leukocytes are visible (right). Quantification of leukocytes rolling **(C)** or sticking **(D)** to the endothelium of pial venules. For a better visual representation of the full-time course of LEIs, the results of the one-hour time point already shown in Figure 1 were added to the current figure (left of the dashed line). One-way ANOVA: **p < 0.01 and ***p < 0.001 vs. sham. Mean ± SD of 12 animals per group.

For the investigation of later time points after cerebral ischemia, animals were allowed to wake up after stroke and were reanesthetized at different time points to perform *in vivo* microscopy (Figure 2A). These experiments demonstrated that the number of rolling and adherent leukocytes in pial venules continued to increase up to five h after reperfusion (Figure 2B). In contrast to the expected crescendo scenario, that is, a constant increase of LEI over time, we observed the maximal number of interactions of leukocytes with the venous endothelium five h after reperfusion (Figures 2C,D). Thereafter, LEI started to decrease and returned to baseline 15 h after reperfusion.

**Figure 3:**
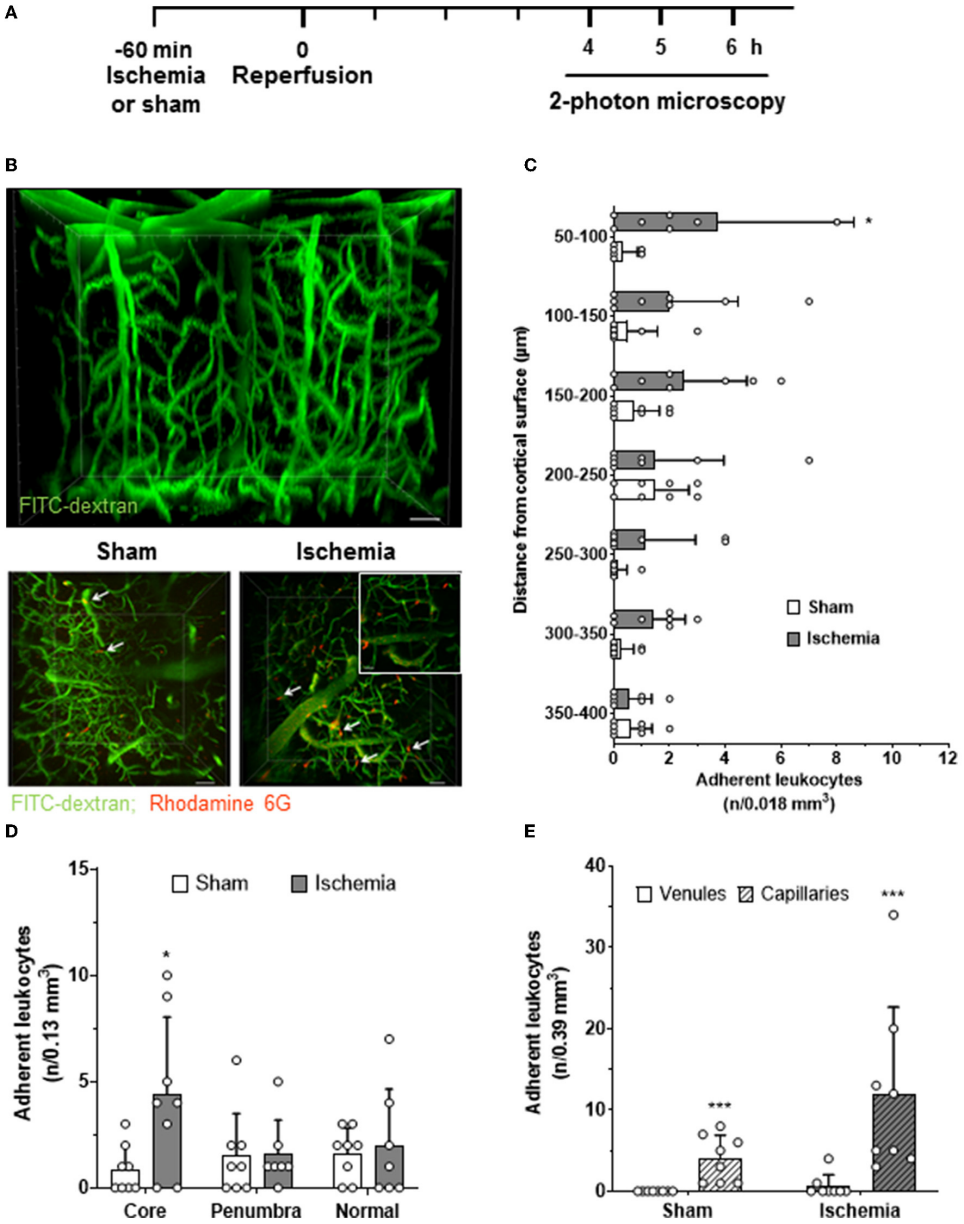
Leukocyte adhesion within the cerebral cortex after cerebral ischemia. **(A)** Experimental design. Animals were reanesthetized 4–6 h after cerebral ischemia (solid line). Three 3D images (0.13 mm^3^ each) from the ischemic core, the ischemic penumbra, and normally perfused brain tissue were acquired. **(B)** Representative 3D reconstruction of the cerebral microcirculation assessed by 2-photon microscopy (upper panel; size bar: 20 μm). Maximal intensity projections of image stacks obtained five h after sham surgery or cerebral ischemia (ischemic core; lower panel; size bar: 40 μm). Leukocytes (red) plugged capillaries (magnification image lower right image; size bar: 20 μm) in sham and in the ischemic core of ischemic animals (white arrows). **(C)** The number of leukocytes observed in cerebral vessels as a function of tissue depth (n/one of the seven tissue volumes, i.e., 0.13/7 = 0.018 mm^3^). **(D)** The number of adherent leukocytes in the infarct core, the ischemic penumbra, and the adjacent normal tissue. **(E)** The number of adherent leukocytes in parenchymal venules and capillaries in all three investigated image stacks (*n*/3 × 0.13 = 0.39 mm^3^). *t*-test: *p < 0.05, ***p < 0.001. Mean ± SD; *n* = 7–8 animals per group.

To investigate the interactions of leukocytes with the vascular wall of intraparenchymal vessels, we imaged penetrating arterioles, capillaries, and venules in the cerebral cortex at a depth of 50–400 μm 4–6 h after reperfusion by *in vivo* 2-photon microscopy (Figure 3A). This time window was chosen to match the peak of LEI in pial vessels. Two-photon microscopy is much slower than EFM (5 vs. 50 Hz), but allows reconstruction of the whole vascular tree of the upper layers of the cerebral cortex in 3D (Figure 3B, upper panel). Using this approach, we detected a significantly increased number of leukocytes in deep cortical vessels five h after reperfusion (Figure 3B, lower panels). Interestingly, most leukocytes were observed in the upper cortical layers and their number decreased toward deeper cortical areas (Figure 3C; *p* < 0.05). Most adherent leukocytes were present in the region of interest located within the ischemic core, whereas no increased leukocyte adherence was observed in the ischemic penumbra or in the healthy brain (Figure 3D; *p* < 0.05). When investigating in which vascular bed leukocyte adhesion occurred, we realized that almost all leukocytes got stuck in capillaries and almost no interaction took place in venules (Figure 3E; *p* < 0.001) or arterioles (data not shown). Interestingly, leukocytes plugged a small number of capillaries also in sham-operated, nonischemic control mice, suggesting that plugging of capillaries by leukocytes may represent a physiological process (Figure 3E; *p* < 0.001). Ischemia and reperfusion increased the number of plugging leukocytes by 3-fold; however, in absolute numbers, capillary plugging remained on a very low level. Only twelve cells were found in three regions of interest with a total volume of 0.39 μm^3^, that is, three times the volume shown in Figure 3B, whereas already three cells were found under control conditions (Figure 3E; *p* < 0.001).

So far, our findings suggest that following cerebral ischemia, leukocytes undergo an only transient interaction with cerebral vessels. By far, the most interaction occurs in pial venules, whereas only very few leukocytes occlude cortical capillaries.

### Time Course of Neuronal Cell Death and Leukocyte Recruitment in the Area Observed by Intravital Microscopy

**Figure 4:**
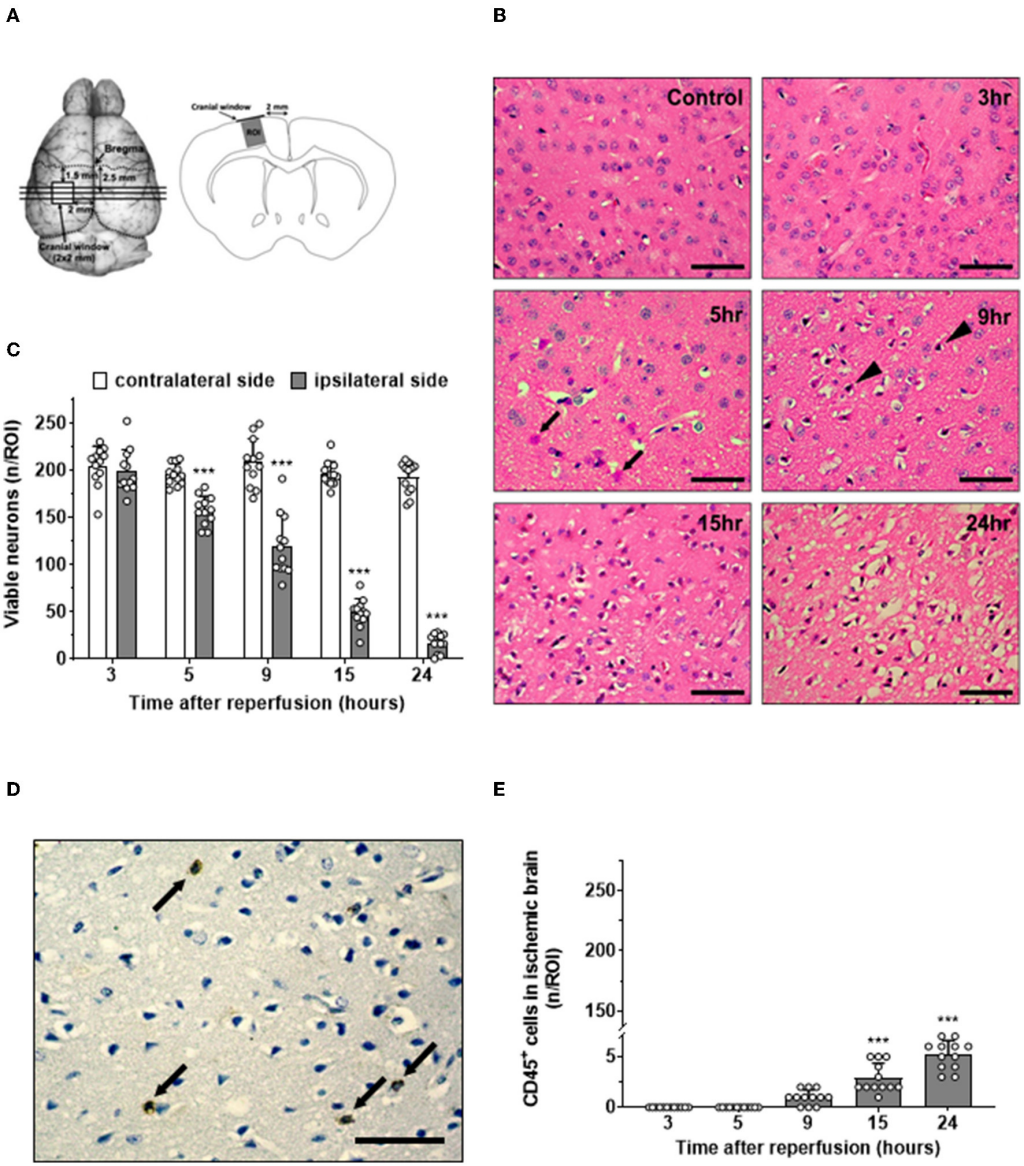
Time course of neuronal cell death and leukocyte migration after ischemic stroke. **(A)** Schematic drawings showing the position of the three coronal sections used for analysis in relation to the imaging windows (left). ROIs for analysis were located in the cerebral cortex just underneath the center of the cranial window (right). **(B)** H&E staining in a sham-operated mice (control) and at different time points after cerebral ischemia (size bar: 50 μm). In the ischemic side, eosinophilic cells started to appear after 5 h (arrow). After 9 h, some cells showed pyknotic nuclei (arrowhead). **(C)** Quantification of healthy appearing neurons. **(D)** Leukocytes (arrows) in ischemic tissue 24 h after reperfusion assessed by CD45 staining (brown; size bar: 50 μm). **(E)** Time course of the number of leukocytes in cerebral cortex 3–24 h after ischemia–reperfusion (adjacent sections to B). One-way ANOVA: ***p < 0.001 vs. previous time point. Mean ± SD. *n* = 12/group.

**Figure 5:**
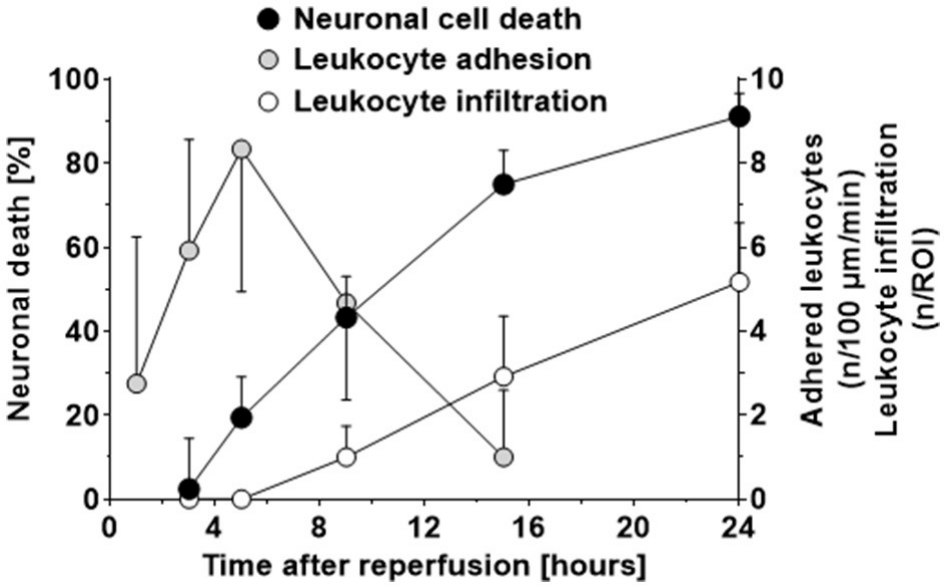
Time course of neuronal cell death, adhesion of leukocytes to postcapillary venules, and infiltration of leukocytes into ischemic brain tissue after cerebral ischemia. The first significant loss of neurons in the ischemic penumbra was observed five h after reperfusion from cerebral ischemia. Adhesion of leukocytes to pial postcapillary venules preceded neuronal cells death and peaked just at the moment when neuronal cell death started to become evident, that is, five h after reperfusion. Leukocytes invaded the ischemic penumbra in low numbers (2–3 leukocytes per 100 neurons) nine h after reperfusion, that is, at least 4 h after neuronal cell death already started to occur. These data indicate that leukocytes invade ischemic brain tissue, but only in very low numbers and too late to be responsible for neuronal cell death.

After defining the complete time course of the interaction of leukocytes with the cerebrovascular endothelium following cerebral ischemia, we aimed to investigate the relationship between this process, transmigration of leukocytes into the brain, and neuronal cell death. Since in the past, most studies that addressed similar aims investigated different tissue volumes by histology and *in vivo* microscopy, we took special care to count leukocytes or CD45-positive cells and healthy neurons in exactly the same tissue volume we investigated by *in vivo* microscopy (Figure 4A). Further, we paid special attention to investigate brain tissue at risk for delayed ischemic damage. Therefore, we counted the number of healthy appearing neurons at the dorsal edge of the MCA territory, the area of the brain known to be affected by delayed ischemic injury. When investigating this area of the cerebral cortex, we observed no neuronal cell loss in control animals and almost no damage 3 h after reperfusion. Thereafter, however, we detected increasing damage including vacuolization of the neuropil, eosinophilic “red neurons” (Figures 4B, 5h, arrows), pyknotic nuclei (Figures 4B, 9h, arrowheads), and an overt loss of neurons with a normal plasma-to-nucleus ratio up to 24 h after reperfusion. When quantifying these changes, we found no loss of viable neurons three h after reperfusion and a constantly decreasing number of viable neurons until 24 h after reperfusion, suggesting that we indeed investigated cerebral tissue affected by delayed ischemic neuronal cell death, that is, the area of the brain potentially salvageable after ischemic stroke (Figure 4C).

To investigate the relationship and time course of delayed neuronal cell death and leukocytes recruited to ischemic tissue, we stained sections adjacent to the ones that are used to quantify neuronal death for leukocytes using an anti-CD45 antibody. Leukocytes were readily identified by their intense brown color, were separated from each other, and had not particular spatial relationship with normal-looking or pyknotic nuclei 24 h after reperfusion (Figure 4D, arrows). When quantifying the number of leukocytes in the area of delayed ischemic neuronal injury over time, we did not find a single leukocyte 3 or 5 h after reperfusion (Figure 4E). Nine h after reperfusion, one or two leukocytes per ROI were found, and only later than 15 h after reperfusion, at time points when most neuronal cell death already occurred, significant numbers of leukocytes were present in the ischemic brain. Even at these late time points, the absolute number of leukocytes was very low, that is, in a region of interest containing 200 neurons (Figure 4C), a mean of 5.2 ± 1.4 leukocytes were counted (Figure 4E).

When plotting the data obtained so far, that is, neuronal cell death, leukocyte adhesion to the cerebrovascular endothelium, and leukocytes infiltrating the ischemic brain after reperfusion over time (Figure 5), it became apparent that 1) LEI in pial venules (Figure 5; gray circles) occurs before neuronal cell death occurred (Figure 5; black circles) and 2) transmigration of leukocytes into the tissue of risk for infarction (Figure 5; white circles) took place only after neuronal cell death already occurred. Based on this observation, we hypothesized that the only meaningful leukocyte-related mechanisms that can be responsible for ischemic tissue injury are leukocyte rolling or adhering to pial venules.

### Effect of LEI Inhibition on Neuronal Cell Death and Leukocyte Infiltration

**Figure 6:**
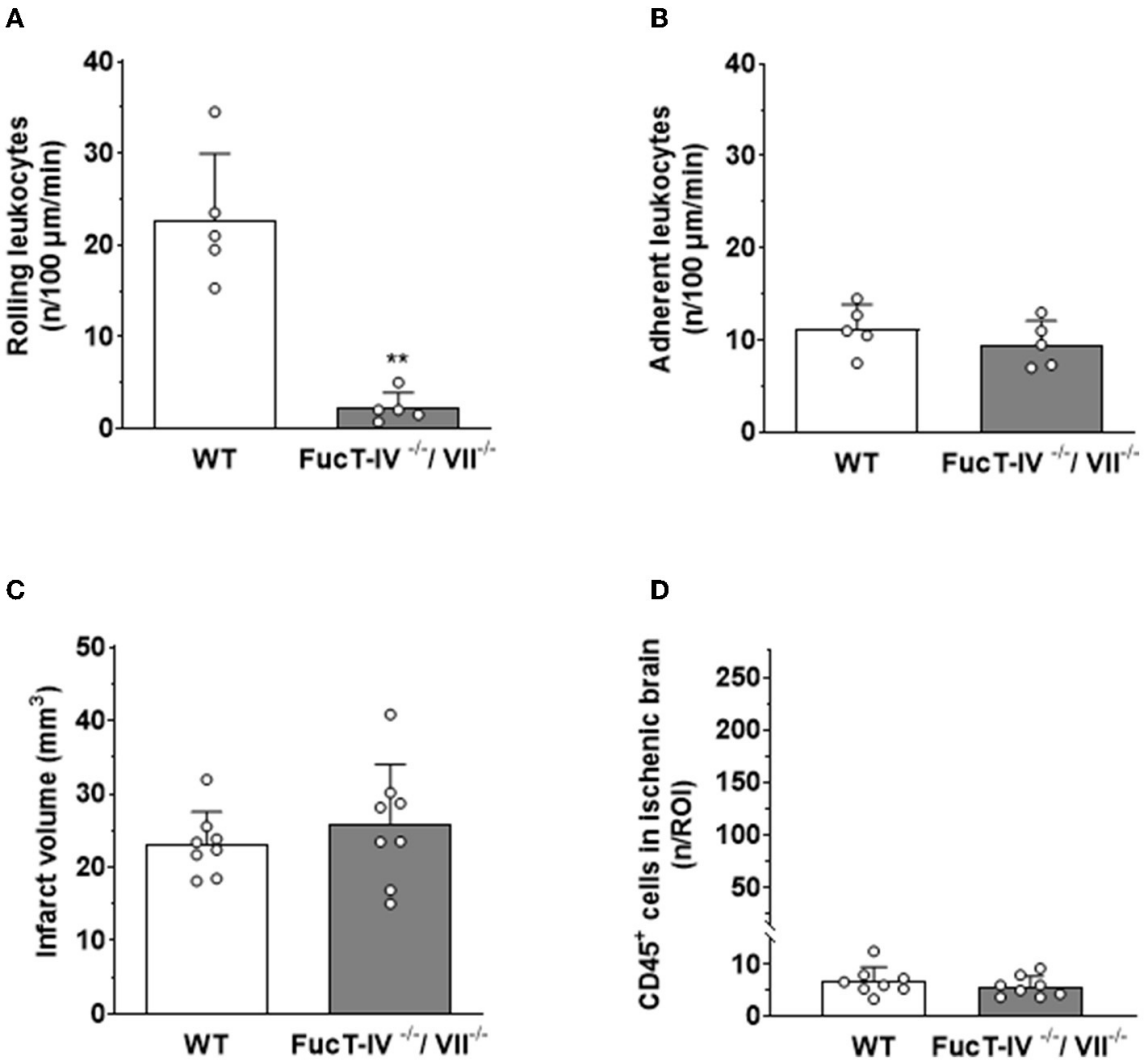
LEIs, leukocyte migration, and tissue damage after cerebral ischemia in mice with nonfunction selectins. FucT IV- and VII-deficient mice lack functional selectins. **(A)** Lack of selectin function significantly reduced the number of leukocytes rolling along pial postcapillary venules, but **(B)** had no effect on firm leukocyte adhesion 5 h after reperfusion. Hence, selectins are not necessary for firm adhesion of leukocytes to the endothelium of pial postcapillary venules after cerebral ischemia. **(C)** Lack of functional selectins had no effect on ischemic brain damage or **(D)** the number of leukocytes migrating into the brain 24 h after cerebral ischemia. Mann–Whitney *U* test: **p < 0.01 vs. control. Mean ± SD; *n* = 5–8 animals per group.

To investigate this hypothesis, we aimed to reduce or inhibit the interaction of leukocytes with pial venules. The first step of LEIs is rolling along the activated endothelium mediated by selectins. To inhibit this interaction, we used mice deficient for fucosyltransferase IV and VII (FucT IV/VII), an enzyme, which adds sugar moieties to the protein backbone of all selectins. Lack of FucT IV/VII therefore almost completely inhibits physiological and pathological leukocyte rolling (74). After subjecting these mice to focal cerebral ischemia, we could indeed observe a highly significant decrease of rolling leukocytes five h after reperfusion as compared to wild-type mice (Figure 6A). Surprisingly, prevention of leukocyte rolling, that is, the first step in the leukocyte recruitment cascade, had no effect on firm adhesion of leukocytes to the vascular endothelium (Figure 6B); leukocytes adhered directly from the center of the bloodstream to the vessel wall and got stuck to the endothelium after a few intermittent interactions (data not shown). When investigating the infarct volumes in these animals 24 h after cerebral ischemia, we did not observe any difference between wild-type littermates and FucT IV-/VII-deficient mice (Figure 6C), suggesting that leukocyte rolling is not necessary for ischemic tissue damage. The number of leukocytes entering the ischemic brain of FucT IV-/VII-deficient mice was as low as in nontransgenic mice (Figure 6D), indicating that rolling does not play a role in the transmigration of leukocytes into ischemic brain tissue.

**Figure 7:**
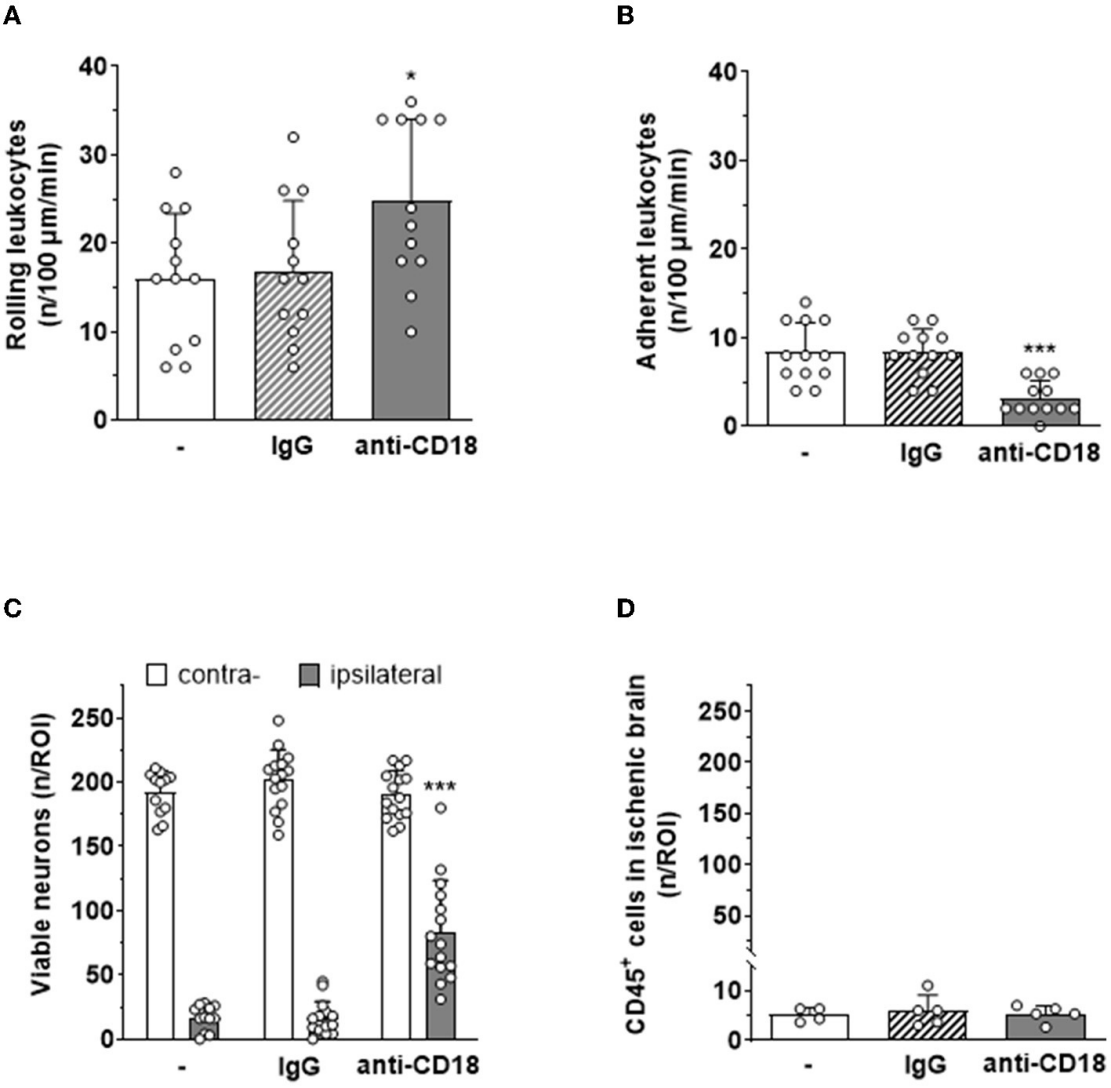
Systemic application of an anti-CD18 antibody reduces leukocyte adhesion to pial postcapillary venules and tissue damage after cerebral ischemia. Blockage of CD18 significantly **(A)** increased rolling (*p < 0.05) and **(B)** decreased adhesion (***p < 0.001) of leukocytes on pial postcapillary venules 5 hours after ischemia–reperfusion. **(C)** Animals receiving an anti-CD18 antibody had five times more viable neurons in their cerebral cortex than animals receiving no treatment or an isotype-matched control IgG antibody 24 h after ischemia–reperfusion (*p* < 0.001). **(D)** Leukocyte migration into ischemic tissue was not affected by anti-CD18 treatment 24 h after ischemia–reperfusion. One-way ANOVA. Mean ± SD; *n* = 5 mice per group.

To investigate the significance of leukocyte adhesion, that is, the second step in the leukocyte recruitment cascade, for ischemic neuronal death, we used a pharmacological approach, that is, an anti-CD18 antibody inhibiting the interaction between leukocyte integrins and endothelial intercellular adhesion molecule 1 (ICAM-1). Application of an anti-CD18 antibody immediately after reperfusion significantly decreased firm adhesion of leukocytes to the endothelium of pial venules 5 hours after reperfusion from cerebral ischemia (Figure 7B), whereas leukocyte rolling was only marginally affected (Figure 7A). When counting surviving neurons in mice receiving the anti-CD18 antibody 24 h after ischemia, we observed a five-fold increase of viable cells in the ischemic brain as compared to untreated mice or animals receiving an isotype-matched control IgG antibody (Figure 7C). The number of leukocytes found within ischemic tissue was not affected by the application of the anti-CD18 antibody (Figure 7D), again suggesting that leukocyte transmigration may not be involved to tissue injury. Very similar findings were observed in ICAM1-deficient mice (Supplementary Figure S2B).

## Discussion

Over the past three decades, a large body of evidence accumulated suggesting that cerebral ischemia elicits a complex and multifaceted acute inflammatory response in the brain (6, 19, 75, 76). Despite the more recently defined roles of microglia and T-lymphocytes (12, 20, 77, 78), mainly circulating polymorphonuclear granulocytes (PMNs) and macrophages are believed to be the major players in this acute process. PMNs are able to promote neuronal cell damage when added to ischemic brain slices (79), are found in tissue and vessels of stroke patients (17–22, 36), and accumulate in the brains of animals subjected to experimental stroke (23–26), and antileukocyte interventions reduce ischemic brain damage in the majority of experimental stroke studies (75, 80). Hence, it seems valid to conclude that leukocytes invading the brain after ischemia contribute to postischemic tissue damage. Despite its simplicity, this concept has been challenged repeatedly mainly on the grounds that even under the most favorable conditions, neurons start to die within the first 12 h after stroke, but significant numbers of leukocytes accumulate in ischemic brain tissue only 24 h after stroke (11, 24, 26, 27, 29, 31, 81). Some authors even suggested that leukocytes may not even fully enter the brain parenchyma after ischemia, but get stuck in the paravascular space (28, 82). Despite these very diverse and almost contradictory observations, a precise and detailed analysis of the tempo-spatial profile of leukocyte accumulation and neuronal cell death has not been performed so far (17). Either leukocytes or neurons were not counted in the very same tissue volume or, more often, leukocytes were analyzed in the infarct core, tissue irredeemably damaged or already dead at the time of analysis. Since leukocytes are involved in the removal of dead tissue, inevitably, positive spatial correlations between the presence of leukocytes and tissue infarction were found, however, without any causal relationship to neuronal cell death (17). The only available option investigating causality between leukocyte invasion and neuronal cell death is to focus on the rim of the ischemic territory, the ischemic penumbra, where tissue is initially viable, but becomes infarcted over time. Only observing the time course of cell death and leukocyte invasion in the very same tissue volume allows to fully appreciate whether these two processes occur at the same time or are temporally and spatially distinct. Using this approach, our current results show for the first time that leukocytes do not invade ischemic tissue in significant numbers before neuronal cell death occurs, that is, within the first 15 h after cerebral ischemia. When comparing the time course of neuronal cell death and tissue damage with the time course of leukocyte invasion, it becomes apparent that leukocytes invade ischemic tissue 3.2 h after neuronal cell death had already occurred. These data strongly suggest that leukocytes invade the ischemic brain too late to be responsible for ischemic neuronal injury. Since we explicitly investigated the ischemic penumbra (as demonstrated by delayed neuronal cell death), our data do not exclude that leukocytes invade already infarcted, that is, not salvageable tissue, as elegantly suggested by others (20, 21, 83), but demonstrate that neuronal cell death and leukocyte invasion into the brain parenchyma are two temporally distinct processes.

**Figure 8:**
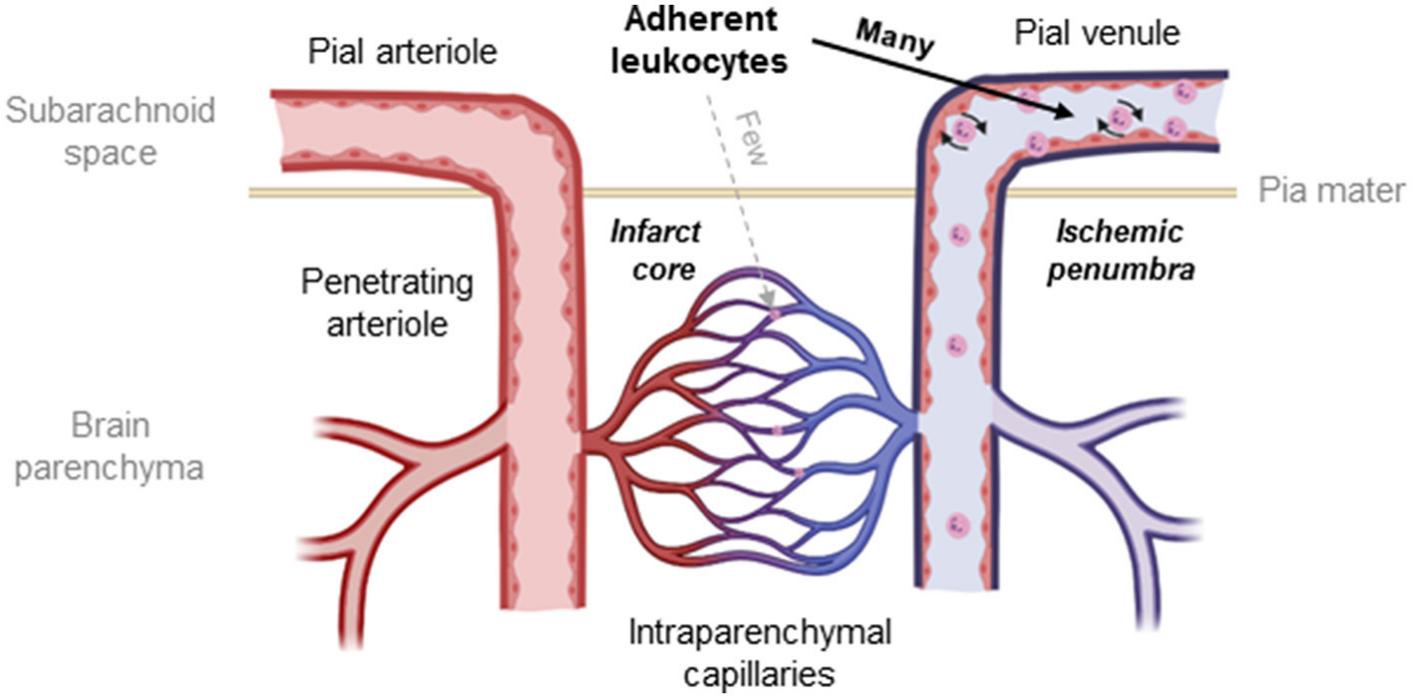
Schematic drawing of the cerebral microcirculation and the site of LEIs after cerebral ischemia. Leukocytes adhere to the endothelium of pial venules and get stuck within the intraparenchymal capillary network after cerebral ischemia. The number of leukocytes blocking capillaries is, however, small, and interactions occur only in the infarct core, the irreversibly damaged tissue that is not amenable to therapy. In contrast, significant numbers of leukocytes adhere to the wall of pial venules in the area of the ischemic penumbra, the main therapeutic target after ischemic stroke.

Despite previous discussions about the role of transmigrating leukocytes for neuronal damage after cerebral ischemia (17, 28, 31, 84, 85) and our current data suggesting that this process does not contribute to ischemic tissue damage, there is still a large number of publications describing neuroprotection by the depletion of leukocytes or by inhibition of leukocyte adhesion to cerebral vessels (17, 33, 52, 57, 86). Hence, it is unclear how the inhibition or prevention of an evidently insignificant pathophysiological process could have a therapeutic effect. One possible explanation for this discrepancy is that these interventions did not only prevent leukocyte-mediated inflammation, but also capillary plugging, a process suggested to be mediated by the same adhesion molecules as inflammatory leukocyte accumulation (87). In fact, capillary plugging by leukocytes has been demonstrated following focal and global cerebral ischemia by radioactive labeling of leukocytes or histopathology (9, 37, 38, 50, 88) and recently by *in vivo* microscopy (33). Further, depletion of leukocytes or inhibition of leukocyte adhesion improved cerebral perfusion and reduced tissue damage (33, 37, 50, 88). Hence, there seems to be a large body of evidence that capillary plugging by leukocytes may also be involved in postischemic brain damage. Interestingly, all studies published so far on this topic addressed either inflammatory leukocyte accumulation or capillary plugging, but never both mechanisms together. Since both processes depend on the presence of leukocytes or the adhesion of leukocytes to the vascular endothelium, inhibiting one of these two mechanisms and investigating only one of the two possible leukocyte-mediated injury mechanisms is not suited to fully understand how leukocytes mediate postischemic tissue damage. For this study, we, therefore, decided to investigate both mechanisms, capillary plugging and inflammatory adhesion of leukocytes to postcapillary venules using the same experimental setup. Using this so far unique approach, we demonstrate that both inflammatory adhesion of leukocytes to postcapillary venules and plugging of cortical capillaries by leukocytes in the ischemic brain occur simultaneously, however, in two almost completely distinct tissue compartments. Inflammation-mediated adhesion of leukocytes occurred almost exclusively in pial venules and was essentially absent in intraparenchymal vessels as also discussed by others (85), whereas capillary plugging was almost exclusively present in intraparenchymal vessels. Thus, we confirmed previous histological and more recent *in vivo* microscopy findings that plugging of capillaries by leukocytes is indeed a feature of ischemic stroke (9, 33, 38). However, in our hands, plugging of capillaries by leukocytes was infrequent and, more importantly, almost exclusively observed in the core of the infarct, that is, unsalvageable or already dead tissue. In other words, our data suggest that leukocytes plug capillaries only in the severely ischemic brain not amenable to therapy, whereas no capillary plugging is observed in the ischemic penumbra, the only salvageable tissue following a stroke (Figure 8).

By inference, the only leukocyte-mediated process left to be responsible for postischemic brain damage is adhesion to pial postcapillary venules. Adhesion of leukocytes to postcapillary venules is a hallmark of inflammation and occurs in all tissues including the brain in a rather prototypic manner: rolling of leukocytes along the vascular endothelium is mediated by endothelial selectins, whereas firm adhesion is mediated by integrins, mainly ICAM-1 and VCAM-1 (87, 89, 90). To underpin the importance of selectins for LEIs after cerebral ischemia, studies in animal models and in patients showed upregulation and elevated plasma levels of E- and P-selectins after stroke (91–95). Blocking selectin function, however, resulted in conflicting results ranging from no to significant neuroprotection in animal models of cerebral ischemia (39, 41, 42, 96, 97). Since issues of genetic background, the influence of selecting deficiency on tissue development, and possible compensation of single selectin knockouts on selectin and integrin expression may have contributed to these conflicting results (98, 99), we decided to use FucT IV-/VII-deficient mice for the current experiments, animals with normal selectin expression but lacking selectin function (59, 74). As expected, the rolling of leukocytes after cerebral ischemia was almost completely blunted in these animals. However, to our surprise, the presumed downstream process of firm adhesion of leukocytes to postcapillary venules was fully maintained, suggesting that postischemic adhesion of leukocytes is not dependent on selectins, but may rather occur due to low shear rates in ischemic tissue as also discussed by others (42). Since FucT IV-/VII-deficient mice showed no neuroprotection after experimental stroke, this unexpected behavior of leukocytes allows us to conclude with so far unavailable level of confidence that selectins and selectin-mediated leukocyte rolling do not play a significant role for postischemic tissue damage.

The last possibility of how leukocytes may induce ischemic tissue injury is by firm adhesion to the endothelium of pial vessels. Therefore, we blocked the interaction between leukocytes and the postcapillary endothelium using an anti-CD18 antibody or mice deficient for ICAM-1. As expected, we observed significantly reduced leukocyte adhesion and consequently increased leukocyte rolling following experimental stroke. Reduced leukocyte adhesion was accompanied by an increase of the number of surviving neurons 24 h after cerebral ischemia; however, the number of transmigrated leukocytes was not affected and remained on a very low level. Based on these quite unexpected results, we conclude that adhesion of leukocytes to postcapillary venules is sufficient for leukocytes to mediate postischemic brain damage.

Inhibition or deletion of ICAM-1 has already been shown by various laboratories to be protective after cerebral ischemia (44–46, 48, 49, 100–102); however, so far, it was believed that blocking adhesion results in reduced transmigration. Our study, however, suggests that adhesion is sufficient to promote neuroprotection and that transmigration occurs too late and involves too few leukocytes to play a role in this process. Thus, our current results offer an explanation for why some previous studies found a discrepancy between neuroprotection and the number of leukocytes found in ischemic brain tissue (17).

Our study may offer some new perspectives, but does of course not resolve all open questions regarding the role of leukocytes for acute ischemic injury. In fact, there are also reports in the literature completely challenging the concept that adhesion of leukocytes plays a role for postischemic brain damage. One prominent example is a recent report elegantly showing that when using knockout mice for stroke experiments, which have a complete deletion of the ICAM-1 gene, no protection is observed (103). However, using animals that mature with a complete lack of ICAM-1, an adhesion molecule with crucial physiological and pathological functions, may well induce compensatory mechanisms which result in alternative adhesion of leukocytes in these animals. Hence, without imaging leukocyte adhesion *in vivo* after ischemic stroke in these animals, a final statement on the role of ICAM-1 for ischemic tissue injury may be premature. Further, mice without any ICAM-1 expression lack alternatively spliced soluble isoforms of the receptor that may bind to circulating leukocytes and prevent their adhesion to ischemic vessels, a process which very much resembles the pharmacological approach used in this study.

The unique advantage of this study is that we visualized the full spatial and temporal dynamics of leukocyte accumulation after cerebral ischemia by *in vivo* imaging. Thereby, we are able to demonstrate that only adhesion of leukocytes to postcapillary pial venules occurs early enough to be involved in ischemic tissue injury. Using a pharmacological and several genetic approaches, we were further able to show that leukocyte adhesion to pial venules is sufficient to trigger postischemic neuronal cell death. Since it is well recognized that leukocytes contribute to tissue repair and regeneration after ischemia (89), our results may explain why inhibition of leukocyte adhesion later than 12 h after stroke resulted in adverse outcomes in clinical studies (104).

In summary, we demonstrate that within the first few hours after reperfusion from cerebral ischemia, leukocytes plug capillaries in the ischemic core and adhere to the endothelium of pial postcapillary venules of the ischemic penumbra, the main therapeutic target following ischemic stroke. Antibody-mediated inhibition of ICAM-1, but not selectins, reduced adhesion of leukocytes to pial postcapillary venules and reduced neuronal cell death, but had no effect on the anyway low number of leukocytes transmigrated into the ischemic brain. By investigating the full spatiotemporal pattern and the molecular mechanisms of leukocyte accumulation to the ischemic brain, our data suggest that early and short-term inhibition of integrin-mediated accumulation of leukocytes may represent a potential therapeutic target for cerebral ischemia. Therefore, our study reinforces the role of leukocytes for secondary brain damage after cerebral ischemia and provides novel insights into the so far not fully understood role of leukocytes for neuronal cell death after ischemic stroke.

## Data Availability Statement

The datasets presented in this study can be found in online repositories. The names of the repository/repositories and accession number(s) can be found in the article/Supplementary Material.

## Ethics Statement

All experimental procedures performed on animals were conducted according to institutional guidelines of the University of Munich and were approved by the Government of Upper Bavaria.

## Author Contributions

NP and HK performed conception and study design. RS, HK, S-WK, and FS carried out surgery, genotyping, neurological testing, and histology. RS, HK, FS, and NP analysed and interpreted the data. RS and HK carried out the statistical analysis. RS, HK, and NP prepared the manuscript. All authors conducted critical revision of the manuscript.

## Funding

The study was funded by the Uehara Memorial Foundation (HK), the Soloz-Zak Research Foundation, the European Union (EVOluTION, project ID 675111), and the Friedrich Baur Foundation (NP), the Munich University's Förderprogramm für Forschung und Lehre (FöFoLe), and the Munich Cluster of Systems Neurology (SyNergy); project ID (EXC 2145/ID 390857198).

## Conflict of Interest

The authors declare that the research was conducted in the absence of any commercial or financial relationships that could be construed as a potential conflict of interest.

## Publisher's Note

All claims expressed in this article are solely those of the authors and do not necessarily represent those of their affiliated organizations, or those of the publisher, the editors and the reviewers. Any product that may be evaluated in this article, or claim that may be made by its manufacturer, is not guaranteed or endorsed by the publisher.
