# Supplementary figures and images for: Adhesion of Leukocytes to Cerebral Venules Precedes Neuronal Cell Death and Is Sufficient to Trigger Tissue Damage After Cerebral Ischemia
Source: Front Neurol. 2022 Jan 24;12:807658. doi: 10.3389/fneur.2021.807658 (PMC8818753; doi:10.3389/fneur.2021.807658)

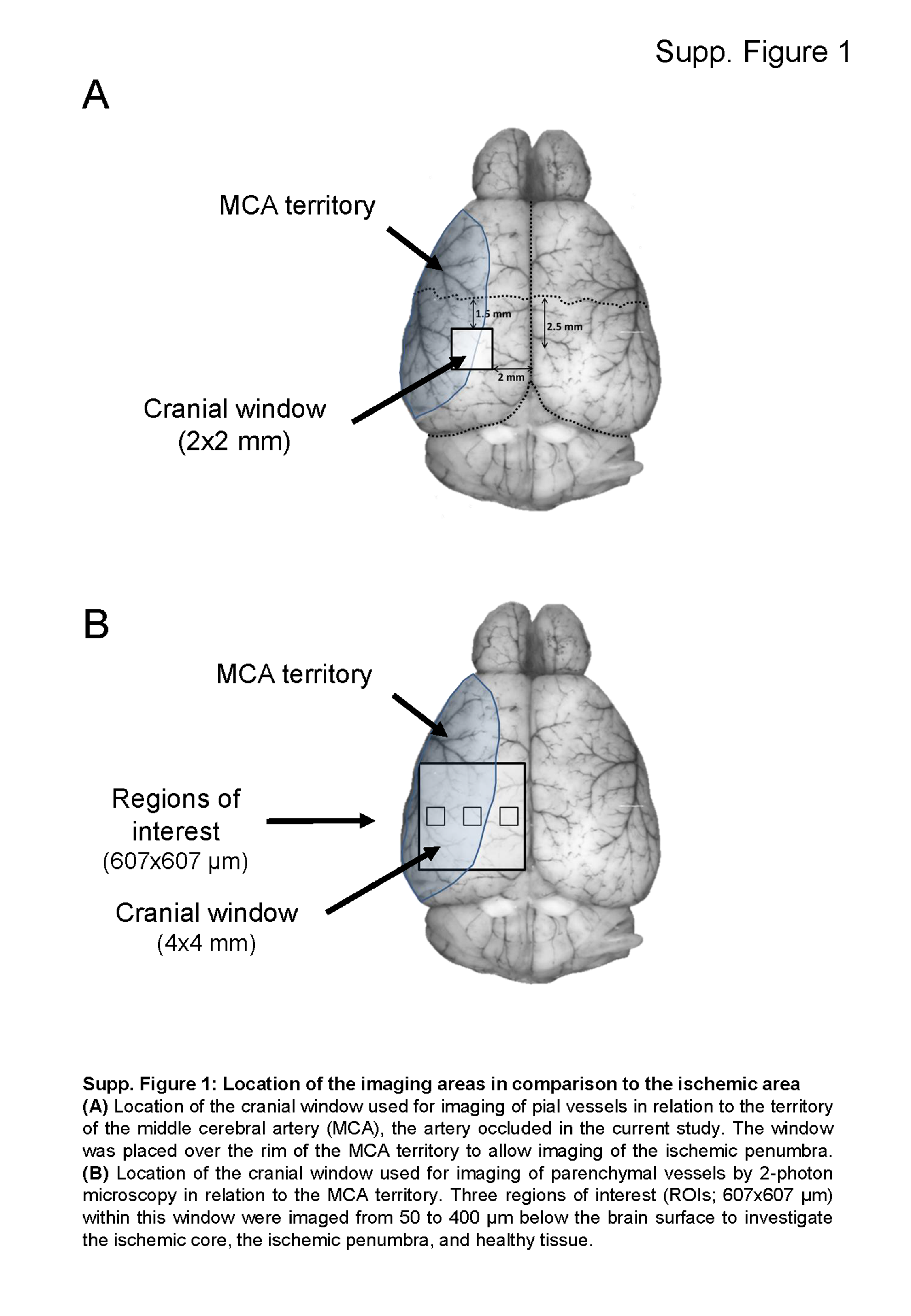

Supplement: Supplementary file 1 [file Image_1.TIFF]

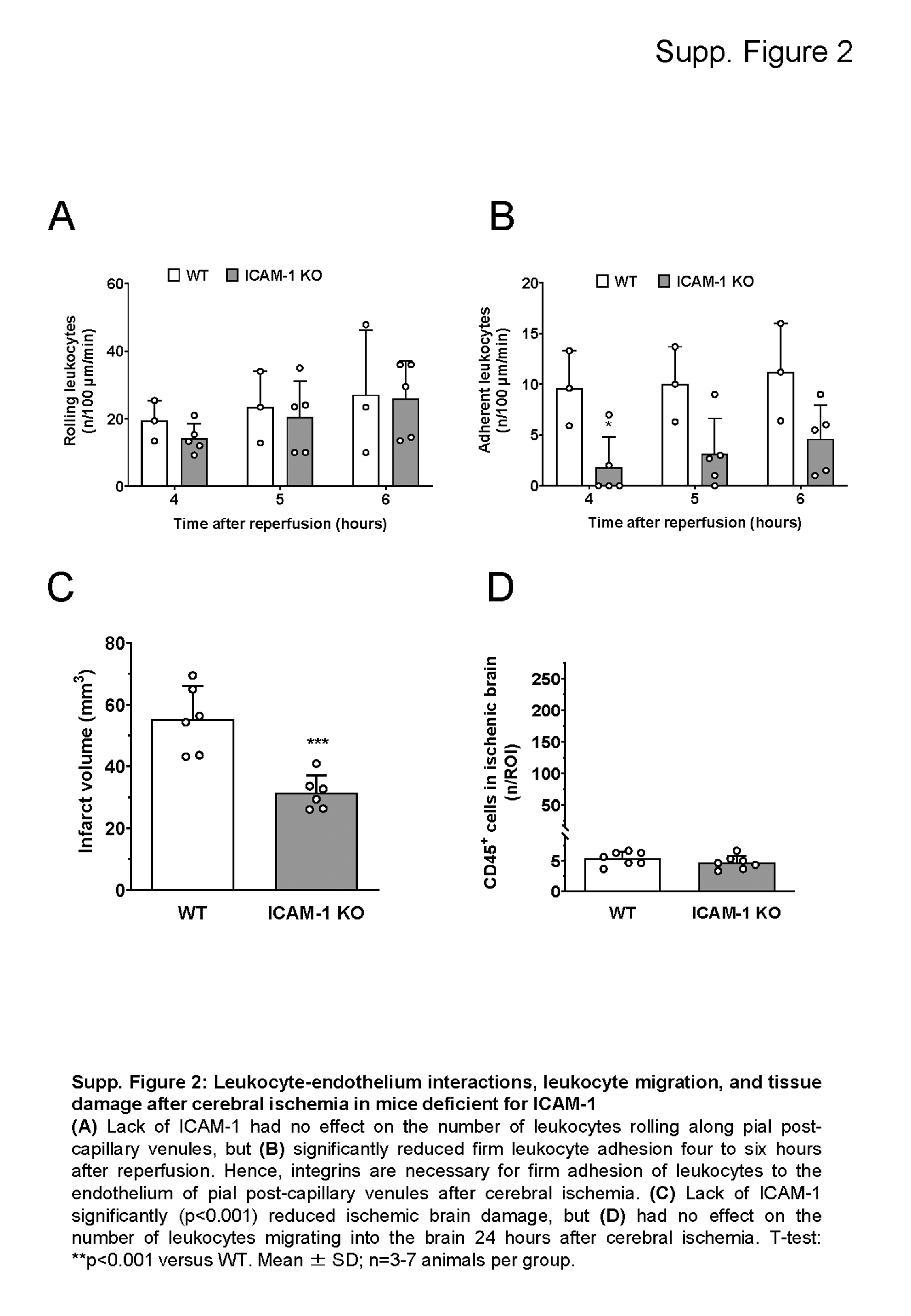

Supplement: Supplementary file 2 [file Image_2.TIFF]

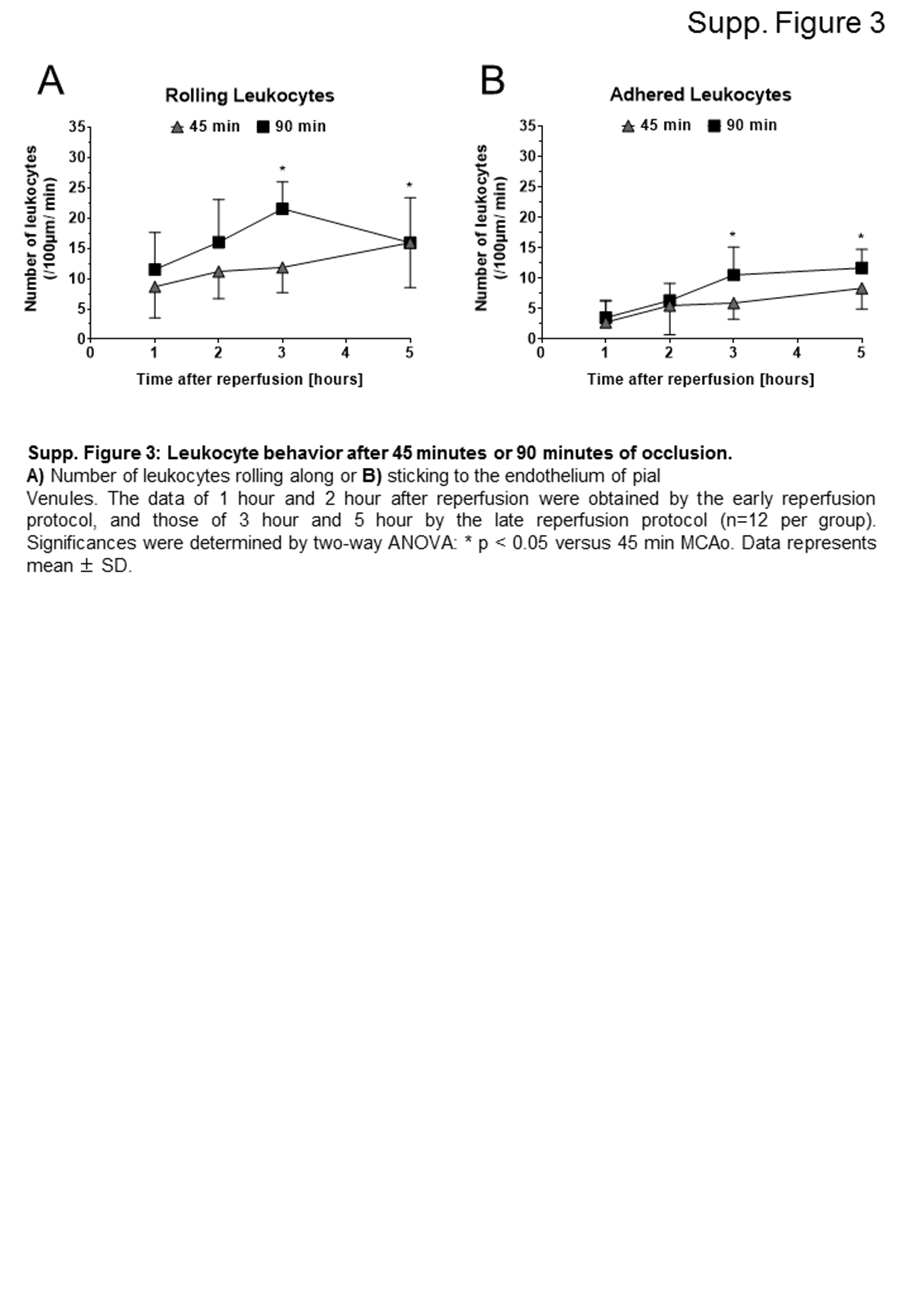

Supplement: Supplementary file 3 [file Image_3.TIF]
